# Association between C-reactive protein-albumin-lymphocyte (CALLY) index and overall survival in patients with colorectal cancer: From the investigation on nutrition status and clinical outcome of common cancers study

**DOI:** 10.3389/fimmu.2023.1131496

**Published:** 2023-03-30

**Authors:** Ming Yang, Shi-Qi Lin, Xiao-Yue Liu, Meng Tang, Chun-Lei Hu, Zi-Wen Wang, Qi Zhang, Xi Zhang, Meng-Meng Song, Guo-Tian Ruan, Xiao-Wei Zhang, Tong Liu, Hai-Lun Xie, He-Yang Zhang, Chen-An Liu, Kang-Ping Zhang, Qin-Qin Li, Xiang-Rui Li, Yi-Zhong Ge, Yu-Ying Liu, Yue Chen, Xin Zheng, Han-Ping Shi

**Affiliations:** ^1^ Department of Gastrointestinal Surgery/Department of Clinical Nutrition, Beijing Shijitan Hospital, Capital Medical University, Beijing, China; ^2^ National Clinical Research Center for Geriatric Diseases, Xuanwu Hospital, Capital Medical University, Beijing, China; ^3^ Key Laboratory of Cancer Foods for Special Medical Purpose (FSMP) for State Market Regulation, Beijing, China; ^4^ Beijing International Science and Technology Cooperation Base for Cancer Metabolism and Nutrition, Beijing, China; ^5^ The Second Affiliated Hospital and Yuying Children’s Hospital of Wenzhou Medical University, Wenzhou, China

**Keywords:** colorectal cancer, prognosis, inflammation, nutrition, immune

## Abstract

**Background:**

Colorectal cancer (CRC) is among the most common malignant cancers worldwide, and its development is influenced by inflammation, nutrition, and the immune status. Therefore, we combined C-reactive protein (CRP), albumin, and lymphocyte, which could reflect above status, to be the CRP-albumin-lymphocyte (CALLY) index, and evaluated its association with overall survival (OS) in patients with CRC.

**Methods:**

The clinicopathological and laboratory characteristics of 1260 patients with CRC were collected from the Investigation on Nutrition Status and Clinical Outcome of Common Cancers (INSCOC) study. Cox regression analysis was performed to assess the association between the CALLY index and OS. A nomogram including sex, age, the CALLY index and TNM stage was constructed. The Concordance Index (C-index) was utilized to evaluate the prognostic value of the CALLY index and classical CRC prognostic factors, such as modified Glasgow prognostic score (mGPS), neutrocyte to lymphocyte ratio (NLR), systemic immune inflammation index (SII), and platelet to lymphocyte ratio (PLR), as well as to assess the prognostic value of the nomogram and TNM stage.

**Results:**

Multivariate Cox regression analyses demonstrated that the CALLY index was independently associated with OS in patients with CRC [Hazard ratio (HR) = 0.91, 95% confidence interval (CI) = 0.87-0.95, *P*<0.001]. The CALLY index showed the highest prognostic value (C-index = 0.666, 95% CI = 0.638-0.694, *P*<0.001), followed by mGPS, NLR, SII, and PLR. The nomogram demonstrated higher prognostic value (C-index = 0.784, 95% CI = 0.762-0.807, *P*<0.001) than the TNM stage.

**Conclusion:**

The CALLY index was independently associated with OS in patients with CRC and showed higher prognostic value than classical CRC prognostic factors. The nomogram could provide more accurate prognostic prediction than TNM stage.

## Introduction

Colorectal cancer (CRC) is one of the most common malignant cancers worldwide and its incidence has been increasing in recent years, posing a significant threat to human health (1, 2). Previous studies have identified several prognostic factors, including neutrophil-to-lymphocyte ratio (NLR), platelet-to-lymphocyte ratio (PLR), systemic immune inflammation index (SII), and modified Glasgow prognostic score (mGPS) (3–5). However, due to their limitations, these factors alone may not provide enough prognostic information to improve survival prediction or select effective treatment strategies. To improve outcomes for patients with CRC, better predictive factors are needed to guide therapy decisions.

Previous studies have shown that the development of CRC is influenced by numerous factors, including the inflammation level, nutritional status, and immune function. The cancer-associated systemic inflammatory response is a critical indicator of tumor progression, and patients with CRC and higher levels of inflammation have a higher risk of death than those with lower levels of inflammation (4, 6). Nutritional status also plays an important role in the prognosis of patients with CRC, with several studies indicating that poor nutrition is linked to poorer overall survival (OS) for patients with CRC (7–9). In addition, good immune function is the main defense against CRC progression. It has been reported that the prognosis of patients with CRC and poor immune function is far worse than that of those with good immune function. Based on the above theories and studies, we believe that an indicator that comprehensively reflects the level of inflammation, nutritional status, and immune function could better predict the prognosis of patients with CRC.

In clinical and past studies, hematological indicators are often used to reflect the inflammation level, nutritional status and immune function of patients with CRC. First, C-reactive protein (CRP) is a common clinical indicator that can reflect the inflammatory levels of patients with CRC (10). Second, serum albumin has been used as an index of nutritional status in clinics for decades (11). Third, lymphocyte count is a traditional biomarker that reflects immune function (12). Finally, we have found that the CRP-albumin-lymphocyte (CALLY) index (a parameter developed by Hiroya Iida et al.) combines CRP, albumin, and lymphocyte, and is a prognostic factor for patients with liver cancer (13).

In this study, we explored the association between the CALLY index and the prognosis of patients with CRC. To determine the superiority and necessity of the CALLY index, we compared its prognostic value to that of classical CRC prognostic factors such as NLR, PLR, SII, and mGPS. Additionally, based on the sex, age, the CALLY index and TNM stage, we developed a nomogram model. We believe that the nomogram could complement the limitations of TNM stage and provide a more accurate prognostic prediction.

## Methods

### Study population

The main methods, results and a specific description of the Investigation on Nutrition Status and Clinical Outcome of Common Cancers (INSCOC) study have been published previously (14). Between January 1, 2012, and October 31, 2020, the INSCOC study enrolled patients who met the inclusion criteria, which included being at least 18 years of age, having a pathological diagnosis of cancer, providing written informed consent, and maintaining consciousness throughout the study. Patients with acquired immune deficiency syndrome, mental or cognitive impairment, or who were organ transplant recipients were excluded from participation. Cases in which patients required more than two hospitalizations during the study were considered single cases.

This was a purely observational study on patients with cancer, without any assignment or intervention. Written informed consent was obtained from all subjects and/or their legal guardians for study participation. The Medical Ethical Review Committees and Institutional Review Boards of the participating registered hospitals approved this study. This study conformed to the Declaration of Helsinki. The study was registered with the Chinese Clinical Trial Registry (http://www.chictr.org.cn) on December 24, 2018 (registration number: ChiCTR1800020329).

**Figure 1:**
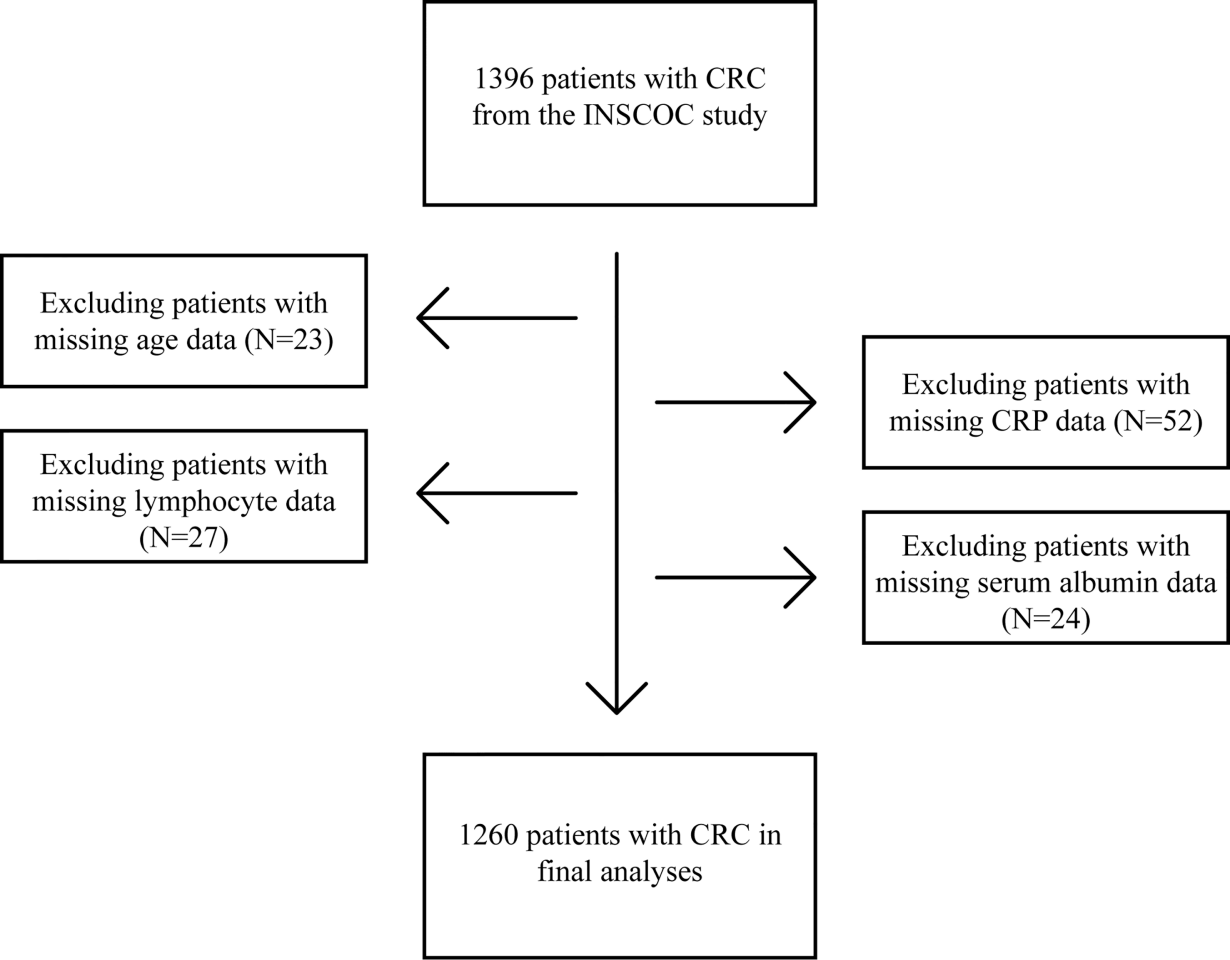
Procedures for selection of study participants with CRC from the INSCOC study. CRC, colorectal cancer; INSCOC, Investigation on Nutrition Status and Clinical Outcome of Common Cancers; CRP, C-reactive protein.

From the INSCOC study, 1396 patients with CRC were enrolled into this study. A total of 136 patients without data on critical variables, including age (23 patients), CRP levels (52 patients), lymphocyte counts (27 patients), and serum albumin levels (24 patients) were excluded from the study (**Figure 1**). Ultimately, 1,260 patients were included in this study.

### Patient characteristics and outcomes

Data on the following demographic and clinicopathological features were collected within 48 h of admission: sex, age, height, weight, smoking status, alcohol consumption, TNM stage, Karnofsky performance status score (KPS), scored patient-generated subjective global assessment (PG-SGA), neutrocyte counts, lymphocyte counts, platelet counts, serum albumin levels, CRP levels, serum creatinine (Scr) levels, blood urea nitrogen (Bun) levels, total cholesterol (Tchol) levels, triglyceride levels, high-density lipoprotein cholesterol (HDL-C) levels, low-density lipoprotein cholesterol (LDL-C) levels, fasting blood glucose (FBG) levels, total bilirubin (Tbil) levels, direct bilirubin (Dbil) levels, aspartate transferase (AST) levels and alanine aminotransferase (ALT) levels. The standard for smoking and drinking were defined as smoking >20 cigarettes in a lifetime and drinking regularly over the past year, respectively. TNM staging followed the guidelines of the American Joint Committee on Cancer. The laboratory tests were performed using the same protocol and reference range nationwide in China. The primary endpoint was patient death due to any reason.

The criteria of mGPS were presented in **Supplemental Table 1**. The method of calculating the CALLY index, NLR, PLR and SII were as follows (13):


CALLY index: Albumin×Lymphocyte÷(CRP×10)

NLR: Neutrocyte÷Lymphocyte

PLR: Platelet÷Lymphocyte

SII: Neutrocyte×Platelet÷Lymphocyte

### Statistical analysis

Data were presented as simple percentages or as medians with interquartile ranges (IQRs). Fisher’s exact test or chi-square tests were used to assess baseline characteristics. Student’s t-tests were used to analyze continuous variables with normal distributions, while the Mann-Whitney test was used for continuous variables with non-normal distributions.

Receiver operating characteristic (ROC) curve was used to determine the cut-off point of the CALLY index, which was 1.47 (**Supplemental Figure 1**). Univariate and multivariate Cox proportional hazards regression models were used to calculate hazard ratios (HR) and their 95% confidence intervals (CI) for each variable in three models (Models 1, 2, and 3). Model 1 was not adjusted for any covariates. Model 2 was adjusted for sex, age, BMI, and TNM stage. Model 3 was adjusted for sex, age, BMI, TNM stage, smoking status, alcohol consumption, KPS and PG-SGA.

Stratified analysis was conducted in each stratifications and sensitivity analysis was conducted after excluding patients with a survival time of less than 1 year to confirm the stability of the association between the CALLY index and OS. Interaction analysis was used to evaluate the interaction between the CALLY index and covariates. Correlation analysis was used to evaluate the relationship between the CALLY index and classical CRC prognostic factors (NLR, PLR, SII and mGPS).

Concordance Index (C-index) and area under the ROC curve (AUC) were used to evaluate the prognostic value of the CALLY index and classical CRC prognostic factors (NLR, PLR, SII and mGPS). C-index, AUC and time-dependent ROC were used to evaluate the prognostic value of the nomogram and TNM stage.

A *P* value less than 0.05 was considered statistically significant. All statistical analyses were performed using R software, version 4.1.1.

## Results

### Patient characteristics

Of all the patients, the median age was 60 years (IQR, 52 to 67 years); the median BMI was 22.58 kg/m^2^ (IQR, 20.55 to 24.84 kg/m^2^); the median CALLY index was 1.35 (IQR, 0.27 to 2.82). 60.9% (767/1260) of the patients were men. 5.6% (71/1260), 20.6% (259/1260), 35.1% (442/1260) and 38.7% (488/1260) of the patients were in stage I, II, III, and IV, respectively. Compared to patients with low CALLY index, patients with high CALLY index had higher proportion of male, TNM stage IV, smoker and drinker, lower proportion of TNM stage I, II and III, older age, higher PG-SGA, neutrocyte counts and platelet counts, and lower BMI, KPS, TC levels, triglyceride levels, HDL-C levels and LDL-C levels. The baseline characteristics were summarized in **Table 1**.

**Table 1:** Characteristics of all patients, patients with high and low CALLY index.

Characteristics	All patients (n = 1260)	Patients with high CALLY index (n = 884)	Patients with low CALLY index (n =376)	*P* value *^e^*
**Sex *^a^* (male)**	767 (60.9)	427 (64.9)	340 (56.5)	0.003
**Age *^b^* (year)**	60.00 [52.00, 67.00]	61.00 [53.00, 68.00]	59.00 [51.00, 66.00]	<0.001
**BMI *^b^* (kg/m^2^)**	22.58 [20.55, 24.84]	22.38 [20.40, 24.68]	22.76 [20.70, 24.97]	0.030
**TNM stage *^a^***				<0.001
**I**	71 (5.6)	34 (5.2)	37 (6.1)	
**II**	259 (20.6)	128 (19.5)	131 (21.8)	
**III**	442 (35.1)	188 (28.6)	254 (42.2)	
**IV**	488 (38.7)	308 (46.8)	180 (29.9)	
**Smoking status *^a,c^* (Yes)**	509 (40.4)	291 (44.2)	218 (36.2)	0.005
**Alcohol consumption *^a,d^* (Yes)**	269 (21.3)	158 (24.0)	111 (18.4)	0.019
**KPS *^b^***	90.00 [80.00, 90.00]	80.00 [80.00, 90.00]	90.00 [80.00, 90.00]	<0.001
**PG-SGA *^b^***	6.00 [2.00, 9.00]	7.00 [4.00, 10.00]	4.00 [2.00, 7.00]	<0.001
**Neutrocyte *^b^* (×10^9^/L)**	3.55 [2.50, 4.97]	4.20 [2.90, 6.00]	3.02 [2.30, 4.00]	<0.001
**Lymphocyte *^b^* (×10^9^/L)**	1.44 [1.08, 1.83]	1.25 [0.90, 1.61]	1.63 [1.30, 2.00]	<0.001
**Platelet *^b^* (×10^9^/L)**	212 [164, 271]	225 [171, 291]	200 [161, 250]	<0.001
**Albumin *^b^* (median [IQR])**	39.75 [35.77, 42.70]	36.80 [33.40, 40.38]	41.75 [39.30, 44.10]	<0.001
**CRP *^b^* (median [IQR])**	3.67 [2.68, 17.20]	16.10 [6.12, 43.72]	2.88 [0.86, 3.20]	<0.001
**Scr *^b^* (μmol/L)**	68.0 [55.1, 80.0]	69.0 [55.0, 80.9]	67.0 [55.5, 78.2]	0.333
**Bun *^b^* (mmol/L)**	5.02 [3.98, 6.22]	5.00 [3.85, 6.36]	5.03 [4.10, 6.17]	0.615
**Tchol *^b^* (mmol/L)**	4.48 [3.86, 5.27]	4.38 [3.69, 5.15]	4.66 [4.06, 5.40]	<0.001
**Triglyceride *^b^* (mmol/L)**	1.35 [1.00, 1.82]	1.30 [0.96, 1.73]	1.39 [1.02, 1.96]	0.011
**HDL-C *^b^* (mmol/L)**	1.16 [0.97, 1.38]	1.12 [0.92, 1.31]	1.22 [1.02, 1.45]	<0.001
**LDL-C *^b^* (mmol/L)**	2.81 [2.26, 3.32]	2.75 [2.15, 3.32]	2.88 [2.34, 3.33]	0.017
**FBG *^b^* (mmol/L)**	5.32 [4.86, 6.13]	5.39 [4.83, 6.46]	5.28 [4.88, 5.87]	0.066
**Tbil *^b^* (μmol/L)**	10.9 [8.3, 15.2]	10.7 [8.0, 15.1]	11.3 [8.6, 15.2]	0.176
**Dbil *^b^* (μmol/L)**	3.0 [2.1, 4.2]	3.2 [2.2, 4.6]	3.0 [2.1, 3.8]	<0.001
**AST *^b^* (U/L)**	22 [17, 29]	22 [17, 30]	22 [18, 29]	0.631
**ALT *^b^* (U/L)**	19 [13, 29]	19 [12, 29]	19 [14, 29]	0.104

CALLY, C-reactive protein–albumin–lymphocyte; BMI, body mass index; KPS, Karnofsky performance status score; PG-SGA, Scored Patient-Generated Subjective Global Assessment; Scr, serum creatinine; Bun, blood urea nitrogen; Tchol, total cholesterol; HDL-C, high-density lipoprotein cholesterol; LDL-C, low-density lipoprotein cholesterol; FBG, fasting blood glucose; Tbil, total bilirubin; Dbil, direct bilirubin; AST, aspartate transferase; ALT, alanine aminotransferase.

^*a*^Categorical variables are presented as number (percentage).

^*b*^Continuous variables are presented as median [interquartile range].

^*c*^The standard is to smoke more than 20 cigarettes in a lifetime.

^*d*^The standard is regular drinking in the past year.

^*e*^The P value was for patients with high and low CALLY index.

### Prognostic role of the CALLY index

Univariate and multivariate Cox regression analyses indicated that the CALLY index was negatively related to the risk of death (**Supplemental Figure 2**). Patients with high CALLY index had a lower death risk (HR = 0.45, 95% CI = 0.36-0.56, *P* <0.001) compared to those with low CALLY index (**Table 2**, **Supplemental Figure 3**). When the CALLY index was divided into 4 quartiles (1^st^ quartile: CALLY index<0.27; 2^nd^ quartile: 0.27≤ CALLY index<1.35; 3^rd^ quartile: 1.35≤ CALLY index<2.82; 4^th^ quartile: CALLY index ≥2.82), patients in the 2^nd^ quartile (HR = 0.69, 95% CI =0.53-0.88, *P* = 0.004), 3^rd^ quartile (HR = 0.46, 95% CI =0.34-0.61, *P*<0.001), and 4^th^ quartile (HR = 0.32, 95% CI = 0.23-0.45, *P*<0.001) had a significantly lower risk of death compared to those in the 1^st^ quartile (**Table 2**).

**Table 2:** Associations between the CALLY index and OS in patients with CRC.

CALLY index	Model 1 *^a^*		Model 2 *^b^*		Model 3 *^c^*	
	HR (95% CI)	*P* value	HR (95% CI)	*P* value	HR (95% CI)	*P* value
**Continues**	0.87 (0.83, 0.91)	<0.001	0.90 (0.86, 0.93)	<0.001	0.91 (0.87, 0.95)	<0.001
**Low *^d^***	Reference		Reference		Reference	
**High *^d^***	0.36 (0.29, 0.44)	<0.001	0.42 (0.33, 0.52)	<0.001	0.45 (0.36, 0.56)	<0.001
**Quartile 1 *^e^***	Reference		Reference		Reference	
**Quartile 2 *^e^***	0.71 (0.56, 0.90)	0.005	0.62 (0.48, 0.79)	<0.001	0.69 (0.53, 0.88)	0.004
**Quartile 3 *^e^***	0.41 (0.31, 0.54)	<0.001	0.40 (0.30, 0.53)	<0.001	0.46 (0.34, 0.61)	<0.001
**Quartile 4 *^e^***	0.25 (0.19, 0.34)	<0.001	0.29 (0.21, 0.40)	<0.001	0.32 (0.23, 0.45)	<0.001
***P* for trend**	0.63 (0.58, 0.69)	<0.001	0.65 (0.59, 0.72)	<0.001	0.68 (0.62, 0.76)	<0.001

CALLY, C-reactive protein–albumin–lymphocyte; OS, overall survival; CRC, colorectal cancer; HR, hazard ratio; CI, confidence interval; KPS, Karnofsky performance status score; PG-SGA, Scored Patient-Generated Subjective Global Assessment.

^*a*^Model 1 was not adjusted for any covariates.

^*b*^Model 2 was adjusted for sex, age, body mass index, and TNM stage.

^*c*^Model 3 was adjusted for sex, age, body mass index, TNM stage, smoking status, alcohol consumption, KPS and PG-SGA.

^*d*^Low:<1.47; High: ≥1.47.

^*e*^Quartile 1:<0.27; Quartile 2: ≥0.27 and<1.35; Quartile 3: ≥1.35 and<2.82; Quartile 4: ≥2.82.

### Stratified, interaction, sensitivity and correlation analyses

**Figure 2:**
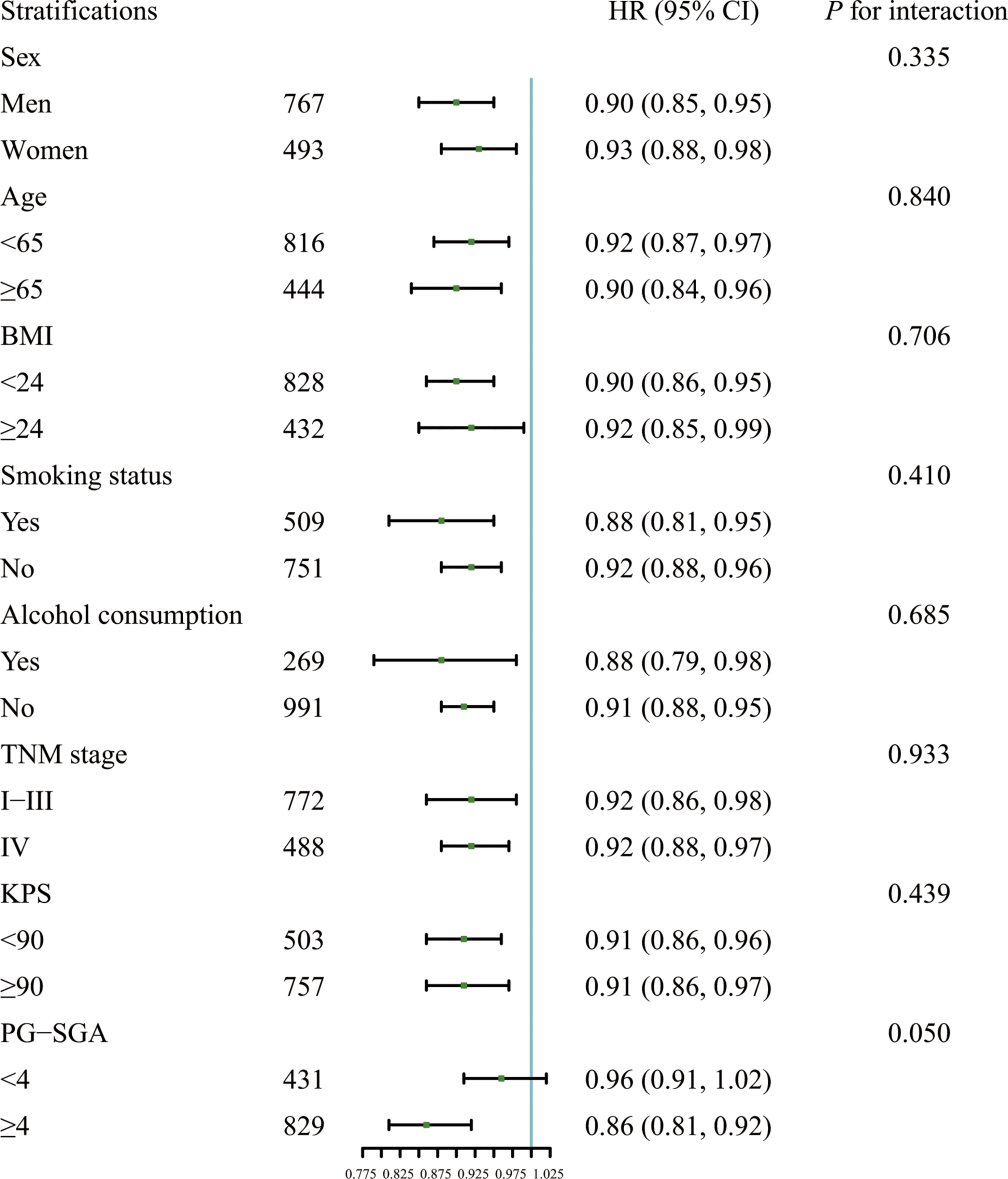
Association between the CALLY index and OS in patients with CRC in different stratifications including sex (men *vs.* women), age (less than 65 years *vs.* 65 years or more), BMI (less than 24 kg/m^2^ *vs.* 24 kg/m^2^ or more), smoking status (Yes *vs.* No), alcohol consumption (Yes *vs.* No), PG-SGA (less than 4 *vs.* 4 or more), tumor stage (I/II/III *vs.* IV), and KPS (less than 90 *vs.* 90 or more). Models were adjusted for sex, age, BMI, smoking status, alcohol consumption, TNM stage, KPS and PG-SGA, but not adjusted for the stratification variable. HR, Hazard ratio; CI, confidence interval; BMI, body mass index; KPS, Karnofsky performance status score; PG-SGA, Scored Patient-Generated Subjective Global Assessment; CALLY, C-reactive protein-albumin-lymphocyte; OS, overall survival; CRC, colorectal cancer.

Results of stratified analysis suggested that the association between the CALLY index and OS was stable in various stratifications, which were divided by the covariates such as sex (men *vs.* women), age (less than 65 years *vs.* 65 years or more), BMI (less than 24 kg/m^2^ *vs.* 24 kg/m^2^ or more), smoking status (Yes *vs.* No), alcohol consumption (Yes *vs.* No), PG-SGA (less than 4 *vs.* 4 or more), tumor stage (I/II/III *vs.* IV) and KPS (less than 90 *vs.* 90 or more) (**Figure 2**). None of the above covariates had an interaction with the CALLY index (all *P* for interaction >0.050).

After excluding patients with a survival time of less than one year, the results of sensitivity analysis showed that a higher CALLY index was significantly associated with a lower risk of death (HR = 0.92, 95% CI =0.88-0.96, *P*<0.001) (**Supplemental Table 2**). Results of correlation analysis showed low correlation between the CALLY index and classical CRC prognostic factors [NLR (*r* = -0.207), PLR (*r* = -0.211), SII (*r* = -0.218) and mGPS (*r* = -0.333)] (**Supplemental Figure 4**).

### Prognostic value of the CALLY index and classical CRC prognostic factors (NLR, PLR, SII and mGPS)

**Figure 3:**
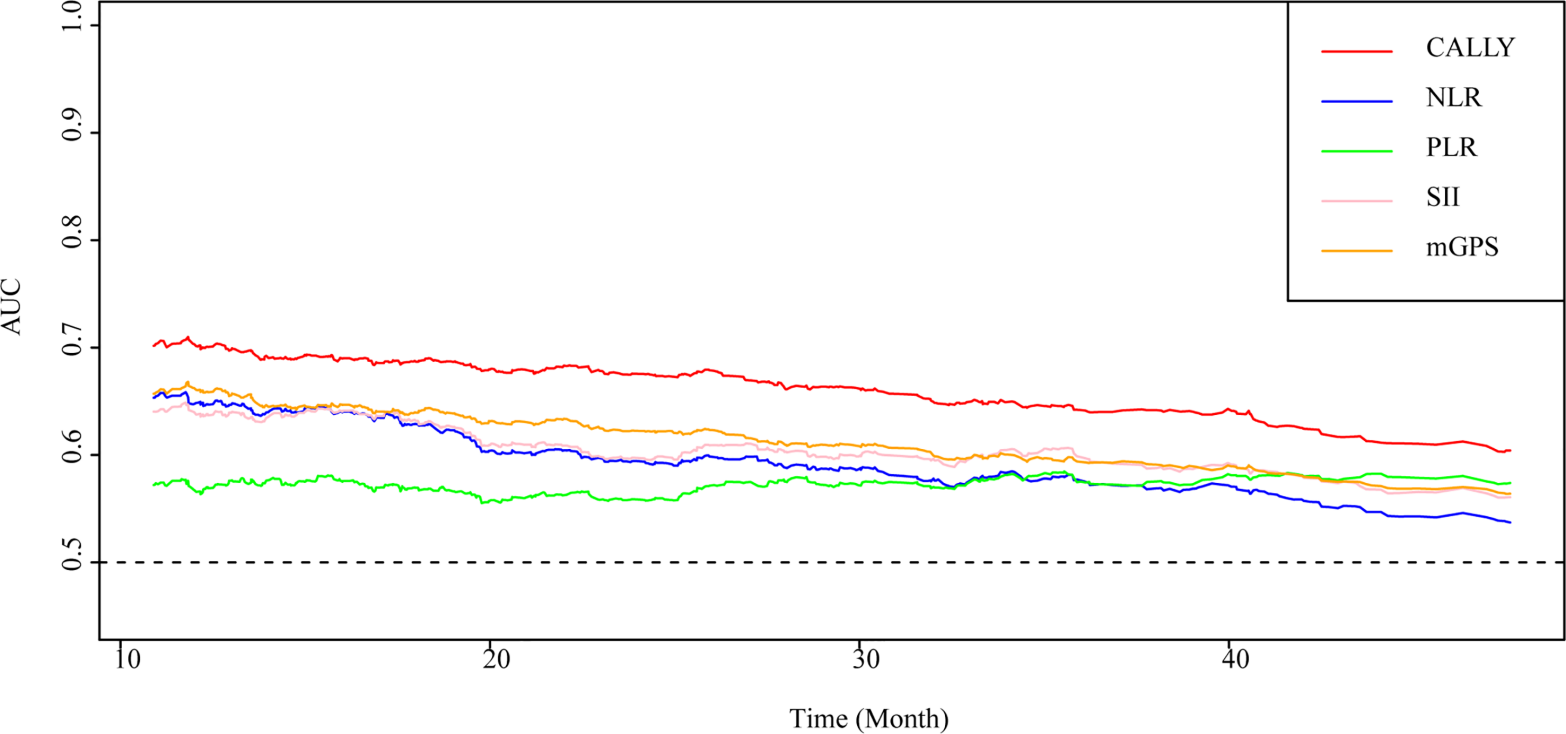
AUC of the CALLY index and classical CRC prognostic factors in patients with CRC. AUC, area under the ROC curve; ROC, receiver operating characteristic; CALLY, C-reactive protein-albumin-lymphocyte; CRC, colorectal cancer; mGPS, modified Glasgow prognostic score; NLR, neutrocyte to lymphocyte ratio; SII, systemic immune inflammation index; PLR, platelet to lymphocyte ratio.

As shown in **Figure 3**, the CALLY index showed the highest prognostic value for patients with CRC, followed by mGPS, NLR, SII and PLR. The C-indices of the CALLY index (C-index = 0.666, 95% CI = 0.638-0.694), mGPS (C-index = 0.623, 95% CI = 0.596-0.650, *P*<0.001), NLR (C-index = 0.614, 95% CI = 0.584-0.644, *P* = 0.001), SII (C-index = 0.611, 95% CI = 0.582-0.641, *P* = 0.001) and PLR (C-index = 0.565, 95% CI = 0.534-0.597, *P*<0.001) were presented in **Supplemental Table 3**.

### Evaluation of the nomogram

**Figure 4:**
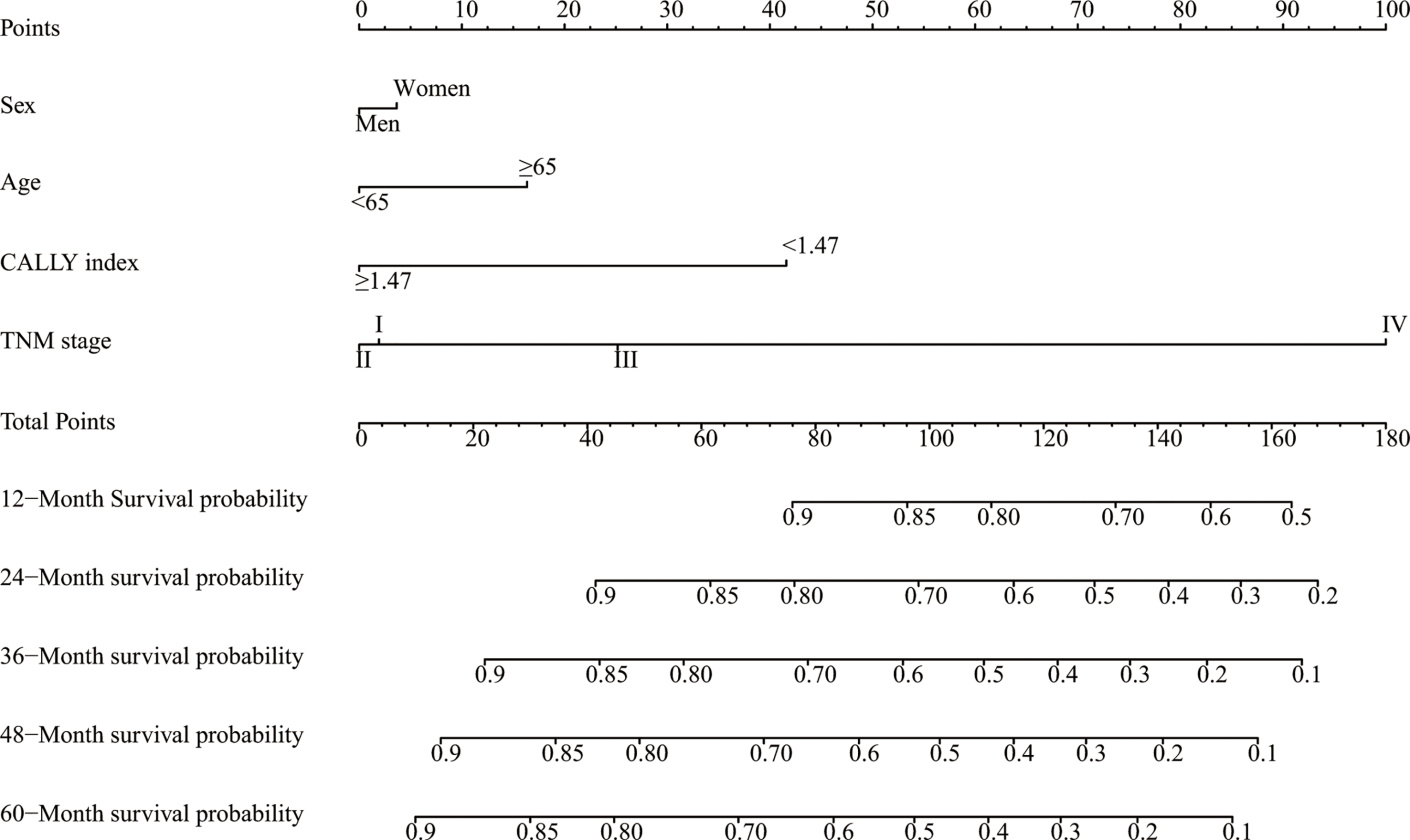
A proposed nomogram for predicting median survival time and survival probability in patients with CRC. Sex, age, the CALLY index and TNM stage were included in the constructed nomogram. To use the nomogram, a line is drawn upward to the Points axis to determine the number of points received for each variable. Sum of these points makes the total points. For total points, a line is drawn from the Total Points axis downward to the survival axes to determine the estimated median survival time and survival probability. CRC, colorectal cancer; CALLY, C-reactive protein-albumin-lymphocyte.

**Figure 5:**
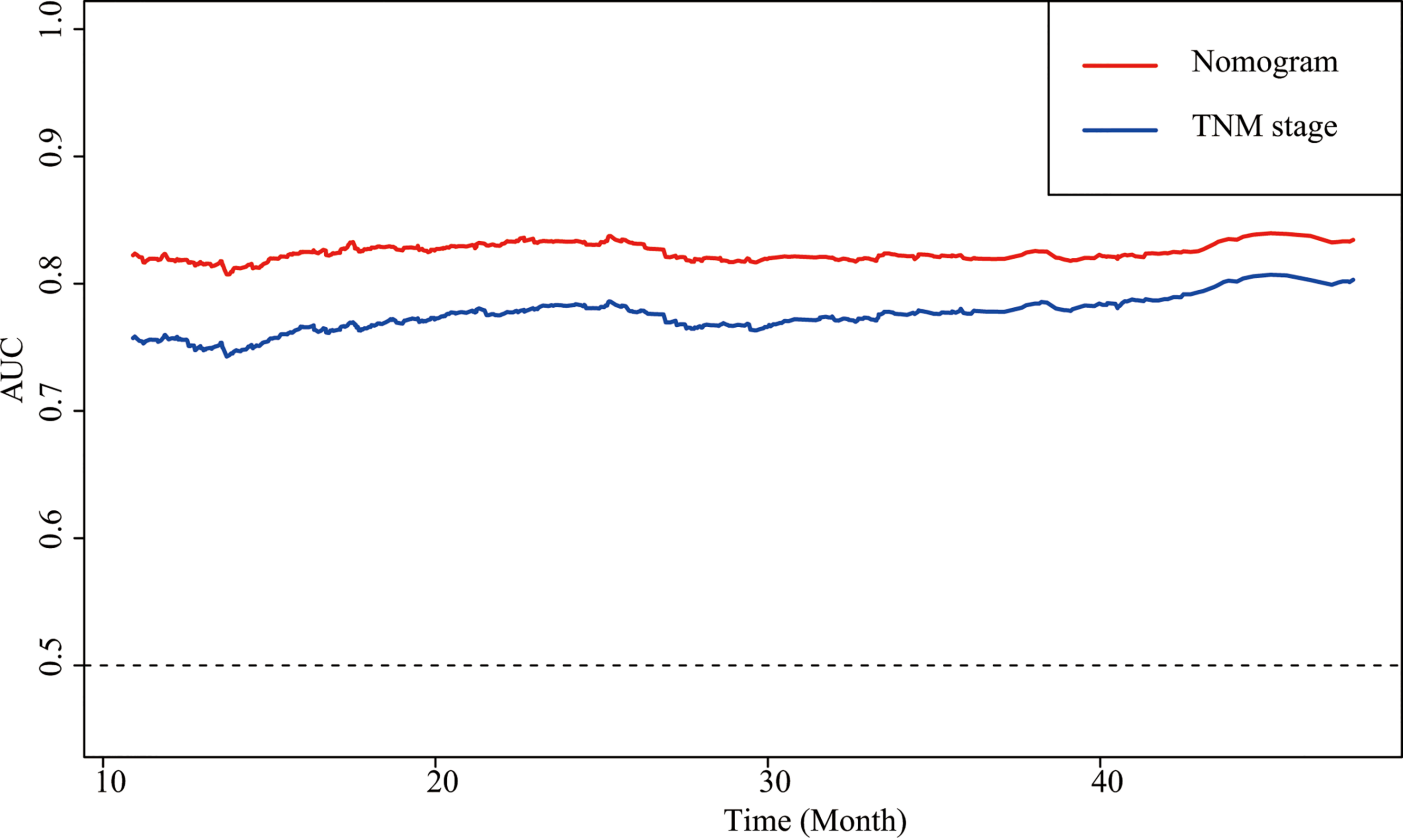
AUCs of the nomogram and the TNM stage in patients with CRC. AUC, area under the ROC curve; ROC, receiver operating characteristic; CRC, colorectal cancer.

Sex, age, the CALLY index and TNM stage were involved the nomogram (**Figure 4**). The calibration curves of the nomogram showed good agreement with the observed outcomes for patients at 1, 2, and 3 years of OS (**Supplemental Figure 5**). The nomogram (C-index = 0.784, 95% CI = 0.762-0.807) showed a significantly higher C-index than TNM stage (C-index = 0.727, 95% CI = 0.704-0.750, *P*<0.001) (**Supplemental Table 4**). As shown in **Supplemental Figure 5**, the nomogram could better predict OS in patients with CRC than the TNM stage (**Figure 5**). Based on the nomogram and the TNM stage, the AUCs of time-dependent ROC curves generated were 81.87% and 75.65% for 1 year, 83.31% and 78.33% for 2 years, and 81.95% and 77.65% for 3 years, respectively (**Supplemental Figures 6A–C**, respectively).

## Discussion

In this study, we identified a specific association between the CALLY index and the prognosis in patients with CRC. We found that an increase in the CALLY index could significantly predict a decrease in the risk of death and that the CALLY index had a higher prognostic value than classical CRC prognostic factors (NLR, PLR, SII, and mGPS). We also developed a nomogram that includes sex, age, the CALLY index and TNM stage to provide accurate predictions. Importantly, this nomogram outperformed the frequently used TNM stage in the clinic when predicting survival outcomes.

The CALLY index consists of CRP, serum albumin and lymphocyte, which can represent inflammation level, nutrition status and immune function, respectively, while inflammation, nutrition, and immunity play important roles in the progression of CRC. Hence, we will discuss the significant association between the CALLY index and OS in patients with CRC from three aspects: inflammation level, nutrition status, and immune function.

CRC is usually accompanied by varying degrees of systemic inflammation, which will influence the incidence and progression of CRC (15). Research showed that inflammation could promote the development and metastasis of CRC through oxidative stress (yields products of oxidative stress such as modified DNA and lipid peroxidation products, and plays an important role in the incidence of CRC), nuclear factor-kappa B (regulating the synthesis of pro-inflammatory cytokines and adhesion molecules), and inflammatory factors such as tumor necrosis factor-alpha, and pro-inflammatory cytokines interleukin (IL) 6 and IL-1 (activating Akt and Wnt, two signaling pathways that was associated with CRC incidence), thus, accelerating disease progression and worsening the survival of patients with CRC (16–24). CRP is a commonly used inflammatory index in clinic (10). Previous studies have shown that elevated CRP represents a more severe inflammatory state, which is associated with a worse prognosis in patients with CRC (5). In our study, a lower CALLY index (representing higher CRP) was associated with a higher risk of death in patients with CRC, which is consistent with research and theories mentioned above.

In addition to inflammation, the roles of nutrition status in the occurrence and development of CRC could not be ignored (7, 8). On one hand, CRC cells affect the absorption and utilization of nutrients through inflammation and metabolic processes, making patients with CRC are prone to malnutrition (25, 26). On the other hand, due to the gastrointestinal symptoms, which affect the appetite and eating, most of the patients with CRC have varying degrees of malnutrition risk (25). Moreover, in conditions such as surgery, trauma, chronic debilitating diseases, and aging, protein synthesis may not occur normally after ingestion of nutrients, leading to anabolic resistance and higher risk of malnutrition in patients with CRC (27). Previous studies have demonstrated that patients with CRC show a weakened response to muscle protein synthesis after injection of a mixture of amino acids (28). Malnutrition in patients with CRC will directly or indirectly affect the prognosis of patients through various ways. First, malnutrition leads to the lack of energy and materials required by the body, gradually unable to maintain basic metabolic activities, eventually resulting in patients with CRC being “starved to death” (29). Second, studies have shown that patients with better nutritional status have higher tolerance for surgery, chemotherapy, and radiotherapy, and the curative effect is better than that of patients with poor nutritional status (30, 31). Serum albumin is a very convenient and intuitive nutritional index (the higher the albumin level, the better the nutritional status) (32). Patients with CRC and hypoalbuminemia are more likely to have unhealthy body composition and poor long-term outcomes (33). Our results indicated that a higher CALLY index (representing higher serum albumin) was associated with a better prognosis in patients with CRC, which is consistent with research and theories mentioned above.

In addition to inflammation level and nutrition status, another factor that must be mentioned that affects the occurrence and development of CRC is immune function. The tumorigenesis process involves different kinds of immune cells. Cancer inhibitory effects occur when lymphocytes are stimulated, such as classically follicular T helper cells, interferon‐ϒ producing T CD8^+^, B lymphocytes and so on (34–40). For example, B lymphocytes could be observed in many cancers and have associations with better prognosis (40). Furthermore, a combination of T and B lymphocytes can induce an effective anti-cancer immune response, as shown B lymphocytes associated with T CD8^+^ lymphocytes shown (40). Additionally, tumor‐infiltrating B lymphocytes have associations with better prognosis in various tumor types (41). In addition to lymphocytes themselves, cytokines secreted by lymphocytes also have anti-tumor effects (42). For example, immunoglobulin E antibodies showed anticancer properties (43). Moreover, based on tumor type, stage, and location, alarmins play different roles in promoting or inhibiting tumor progression (40, 44). In addition, epigenetic changes mediated by microRNA have been shown to influence the development of cancer and immune response (45). In clinical settings, lymphocytes are a representative and commonly used immune index (46). It has also been shown in previous studies that circulating lymphocytes can improve cancer patient outcomes by enhancing cancer immune surveillance, inhibiting cancer cell proliferation, and improving tumor chemoprevention (47, 48). While in the tumor microenvironment, T cell deficiency indicated disruptions in immune regulation and antitumor function (49). Patients with a low lymphocyte count had a shorter survival time than those with a high lymphocyte count (46). In our study, lymphocytes were used as part of the CALLY index. Our results showed that the increment of the CALLY index (increased lymphocyte count) was positively correlated with the improvement of OS in patients with CRC.

Inflammation level, nutrition status and immune function not only affect cancer, but their interactions cannot be ignored, and these interactions would further promote the progression of cancer. Firstly, a higher level of inflammation indicated high levels of cytokines such as IL-1, and IL-6, tumor necrosis factor-alpha and CRP, which greatly accelerates the consumption of nutrition, leading to malnutrition and the progression of CRC (50). Secondly, by activating tumor associated macrophages, myeloid-derived suppressive cells, Cd4^+^Foxp3^+^Treg cells or Th17 cells, inflammation could impair the immune response within tumors, promoting immune deficiency and cancer progression (51). Moreover, given the key roles of nutrition in determining the fate and functions of immune cells, malnutrition could induce an impaired immune response, which have great promotion roles on cancer incidence and progression and finally shorten OS (52). More than that, studies have shown that poor nutritional status could lead to increased levels of inflammation in patients with CRC by gut microbiota, resulting in an increased risk of death (53). To sum up, inflammation, nutrition and immunity interact to produce a complex vicious circle, which further promotes the progress of cancer. We use the CALLY index to combine the representative indicators of inflammation (CRP), nutrition (serum albumin) and immune (lymphocyte), which could not only fully utilize the prognostic value of these three indicators, but also make use of their interaction to comprehensively predict the prognosis.

The results of the stratified and sensitivity analyses indicated that the significant association between the CALLY index and OS in patients with CRC was stable and reliable. The results of the correlation analysis showed low correlation between the CALLY index and classical CRC prognostic factors (NLR, PLR, SII and mGPS), which indicated that the CALLY index can provide clinicians with different and novel prognosis prediction from classical CRC prognostic factors. Moreover, the C-indices and AUC of the CALLY index, NLR, PLR, SII and mGPS clearly showed that CALLY index had the highest prognostic value, which demonstrated the privilege of the CALLY index.

Throughout the world, the TNM stage serves as the most commonly used postoperative staging evaluation system, and is instrumental in treatment and follow-up after surgery (54–56). However, patients with the same TNM stage, often have significant survival heterogeneity, and the TNM stage is inadequate in predicting individual prognoses (57, 58). We believe the reason for the inadequacy of TNM stage is that it only examines the pathology postoperatively and does not consider the basic difference, such as sex and age, and cancer prognostic relative factor, such as inflammation levels, nutrition status and immune function (59, 60). Hence, we developed a nomogram by combining the sex, age, the CALLY index and TNM stage. The C-indices and AUC of the nomogram and TNM stage indicated that the nomogram showed significant higher prognostic value than TNM stage alone. We believe that our nomogram can complement the limitations of TNM stage and help assess the prognosis of patients with CRC more individually and accurately.

To the best of our knowledge, the present study is the first study with the largest number of participants to comprehensively evaluate the association between the CALLY index and survival in patients with CRC. However, the present study has several potential concerns or limitations that are worth mentioning. First, CRP level, albumin level and lymphocyte count were only evaluated at the baseline. More frequent evaluations could enable a more accurate assessment of the association between the CALLY index and the death risk of CRC. Moreover, because the study findings were obtained based on the sample size determined by the INSCOC study conducted from 2012 to 2020, the findings need to be validated in another study with larger and different population. Additionally, due to the scope limitation of laboratory tests used in the INSCOC study, the covariates included in the analysis were limited. Moreover, due to the limitation of external database and the number of study population, we could not conduct external verification and internal verification. In the future, we will conduct more in-depth clinical and laboratory studies with more participants and more confounders to further investigate the underlying mechanisms.

## Conclusion

In summary, CALLY index could be used as independent prognostic factors and showed better prognosis prediction ability than classical CRC prognostic factors (NLR, PLR, SII and mGPS) in patients with CRC. We proposed a nomogram that complemented the shortage of the TNM stage and showed better prognosis prediction ability than the TNM stage. We believe that our nomogram could guide clinicians to facilitate clinical decision-making, individualized treatment, and disease management more accurately and specifically.

## Data availability statement

The raw data supporting the conclusions of this article will be made available by the authors, without undue reservation.

## Ethics statement

The studies involving human participants were reviewed and approved by Medical Ethical Review Committees and Institutional Review Boards of the participating registered hospitals. The patients/participants provided their written informed consent to participate in this study.

## Author contributions

HP-S: Conceptualization, Methodology. MY: Data curation, Writing- Original draft preparation. X-YL: Visualization, Investigation, Data curation. S-QL: Software. MT: Validation, Visualization. C-LH: Writing-Reviewing and Editing. Z-WW: Supervision, Investigation. QZ: Writing-Reviewing and Editing. XiZ: Writing-Reviewing and Editing. M-MS: Writing-Reviewing and Editing. G-TR: Supervision, Investigation. X-WZ: Supervision, Investigation. TL: Software. H-LX: Writing-Reviewing and Editing. H-YZ: Supervision, Investigation. K-PZ: Software. Q-QL: Supervision, Investigation. X-RL: Supervision, Investigation. Y-ZG: Supervision, Investigation. Y-YL: Writing-Reviewing and Editing. YC: Supervision, Investigation. XinZ: Supervision, Investigation. All authors contributed to the article and approved the submitted version.
