# Supplementary material for: Association between C-reactive protein-albumin-lymphocyte (CALLY) index and overall survival in patients with colorectal cancer: From the investigation on nutrition status and clinical outcome of common cancers study
Source: Front Immunol. 2023 Mar 30;14:1131496. doi: 10.3389/fimmu.2023.1131496 (PMC10098202; doi:10.3389/fimmu.2023.1131496)
Supplement: Supplementary file 1 [file DataSheet_1.docx]

Supplementary Material

Association between C-reactive protein-albumin-lymphocyte (CALLY) Index and Overall Survival in Patients with Colorectal Cancer: From the Investigation on Nutrition Status and Clinical Outcome of Common Cancers Study

**Ming Yang^1,2,3,4†^, Shi-Qi Lin^1,2,3,4,5†^, Xiao-Yue Liu^1,2,3,4†^, Meng Tang^1,2,3,4^, Chun-Lei Hu^1,2,3,4^, Zi-Wen Wang^1,2,3,4^, Qi Zhang^1,2,3,4^, Xi Zhang^1,2,3,4^, Meng-Meng Song^1,2,3,4^, Guo-Tian Ruan^1,2,3,4^, Xiao-Wei Zhang^1,2,3,4^, Tong Liu^1,2,3,4^, Hai-Lun Xie^1,2,3,4^, He-Yang Zhang^1,2,3,4^, Chen-An Liu^1,2,3,4^, Kang-Ping Zhang^1,2,3,4^, Qin-Qin Li^1,2,3,4^, Xiang-Rui Li^1,2,3,4^, Yi-Zhong Ge^1,2,3,4,5^, Yu-Ying Liu^1,2,3,4^, Yue Chen^1,2,3,4,5^, Xin Zheng^1,2,3,4^, Han-Ping Shi^1,2,3,4*^**

^1^Department of Gastrointestinal Surgery/Department of Clinical Nutrition, Beijing Shijitan Hospital, Capital Medical University, Beijing, 100038, China.

^2^National Clinical Research Center for Geriatric Diseases, Xuanwu Hospital, Capital Medical University, Beijing, 100053, China

^3^Key Laboratory of Cancer FSMP for State Market Regulation, Beijing, 100038, China.

^4^Beijing International Science and Technology Cooperation Base for Cancer Metabolism and Nutrition, Beijing, 100038, China.

^5^The Second Affiliated Hospital and Yuying Children's Hospital of Wenzhou Medical University, Wenzhou, 325000, China.

^†^These authors have contributed equally to this work and share first authorship.

*** Correspondence:**Han-Ping Shi
[shihp@ccmu.edu.cn](mailto:shihp@ccmu.edu.cn)

**
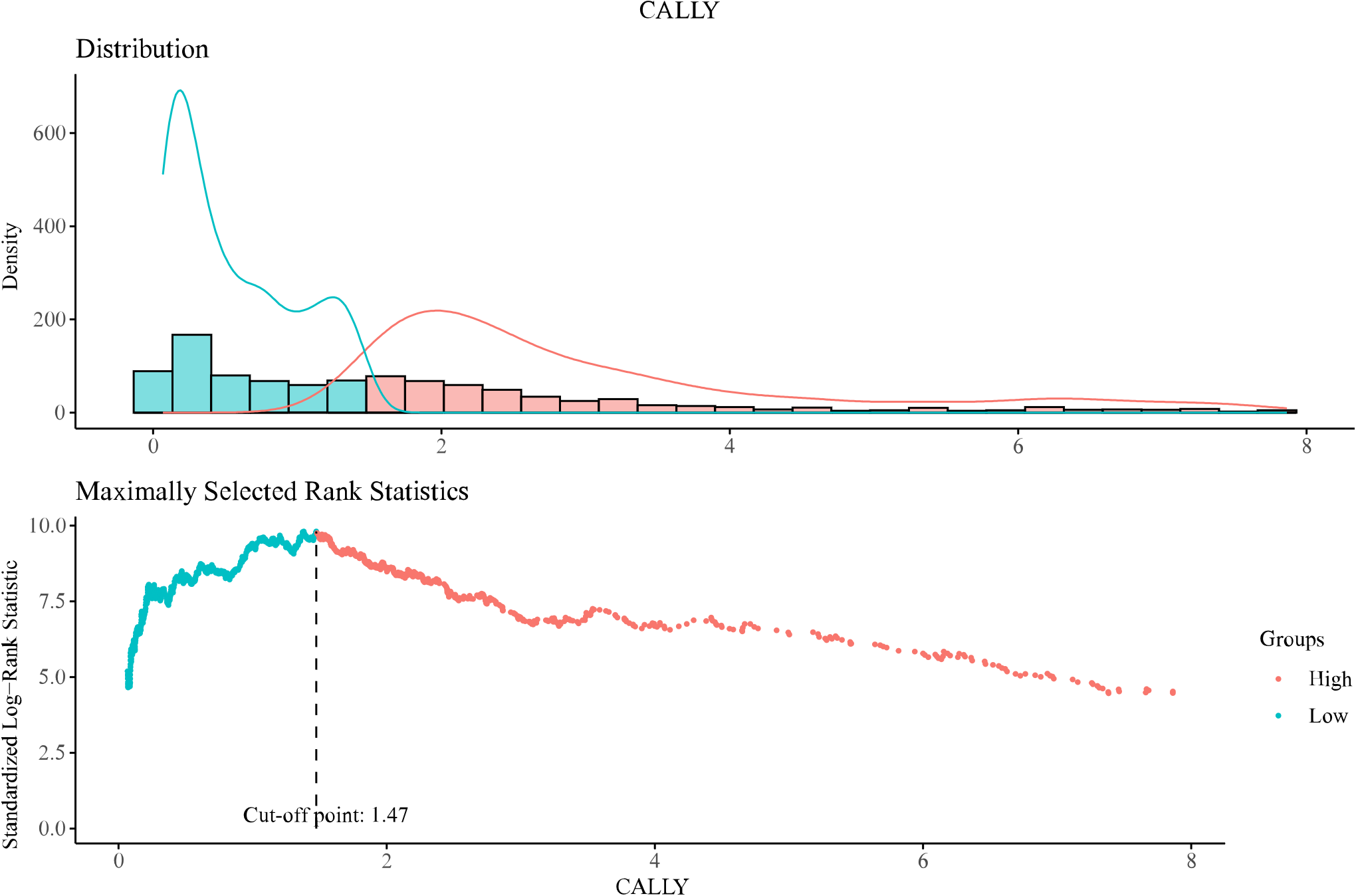
**

**Supplemental Figure 1.** ROC curve for determining the cut-off point of the CALLY index in patients with CRC.

Abbreviation: CALLY, C-reactive protein-albumin-lymphocyte; ROC, receiver operating characteristic; CRC, colorectal cancer.

**
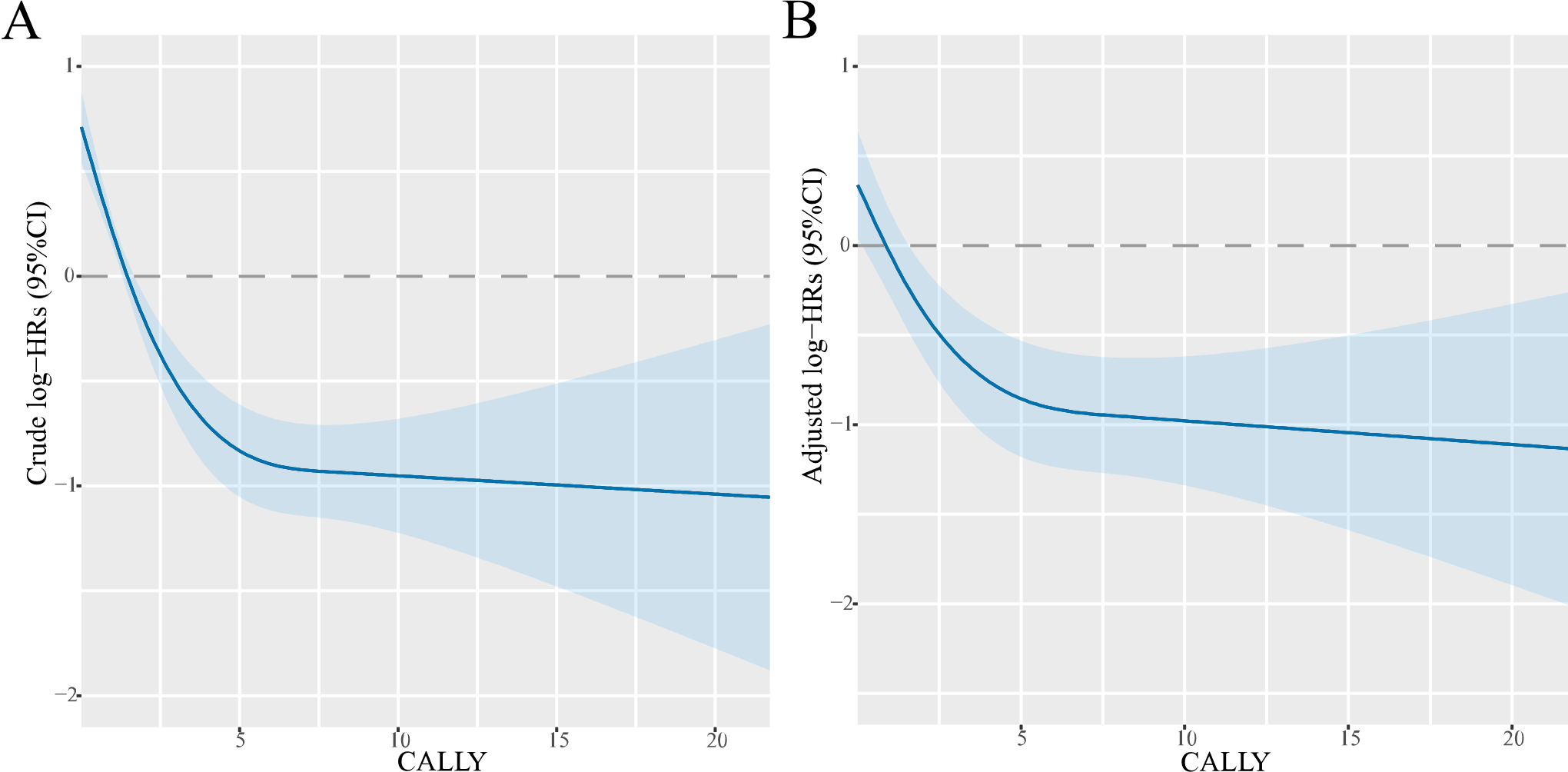
**

**Supplemental Figure 2.** Association between the CALLY index and OS in patients with CRC. Model B was adjusted for sex, age, BMI, TNM stage, smoking status, alcohol consumption, KPS and PG-SGA.

Abbreviation: HR, Hazard ratio; CI, confidence interval; CALLY, C-reactive protein-albumin-lymphocyte; OS, overall survival; CRC, colorectal cancer; BMI, body mass index; KPS, Karnofsky performance status score; PG-SGA, Scored Patient-Generated Subjective Global Assessment.


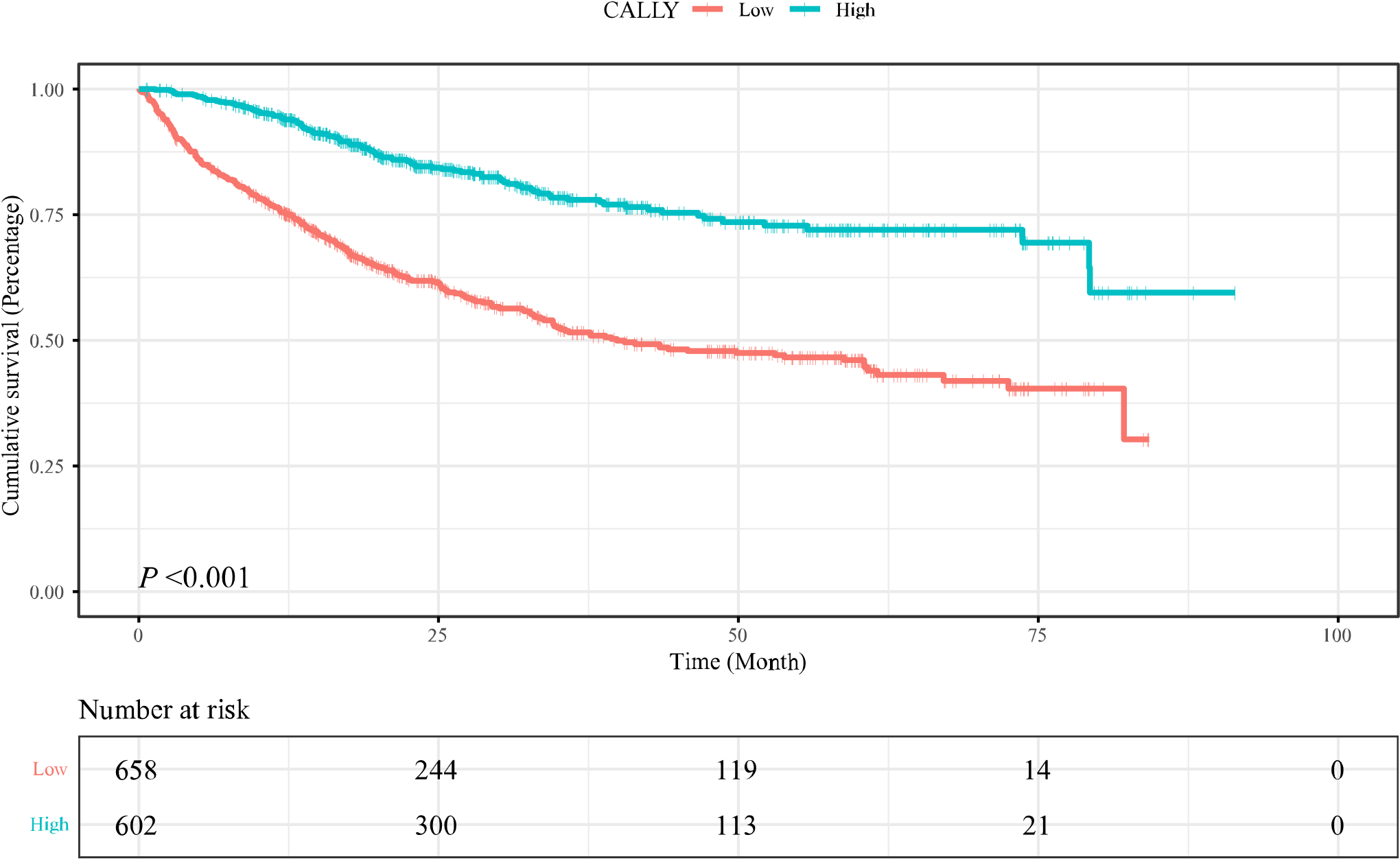


**Supplemental Figure 3.** KM curves showing associations between the CALLY index and OS in patients with CRC.

Abbreviation: CALLY, C-reactive protein-albumin-lymphocyte; KM, Kaplan-Meier; OS, overall survival; CRC, colorectal cancer.


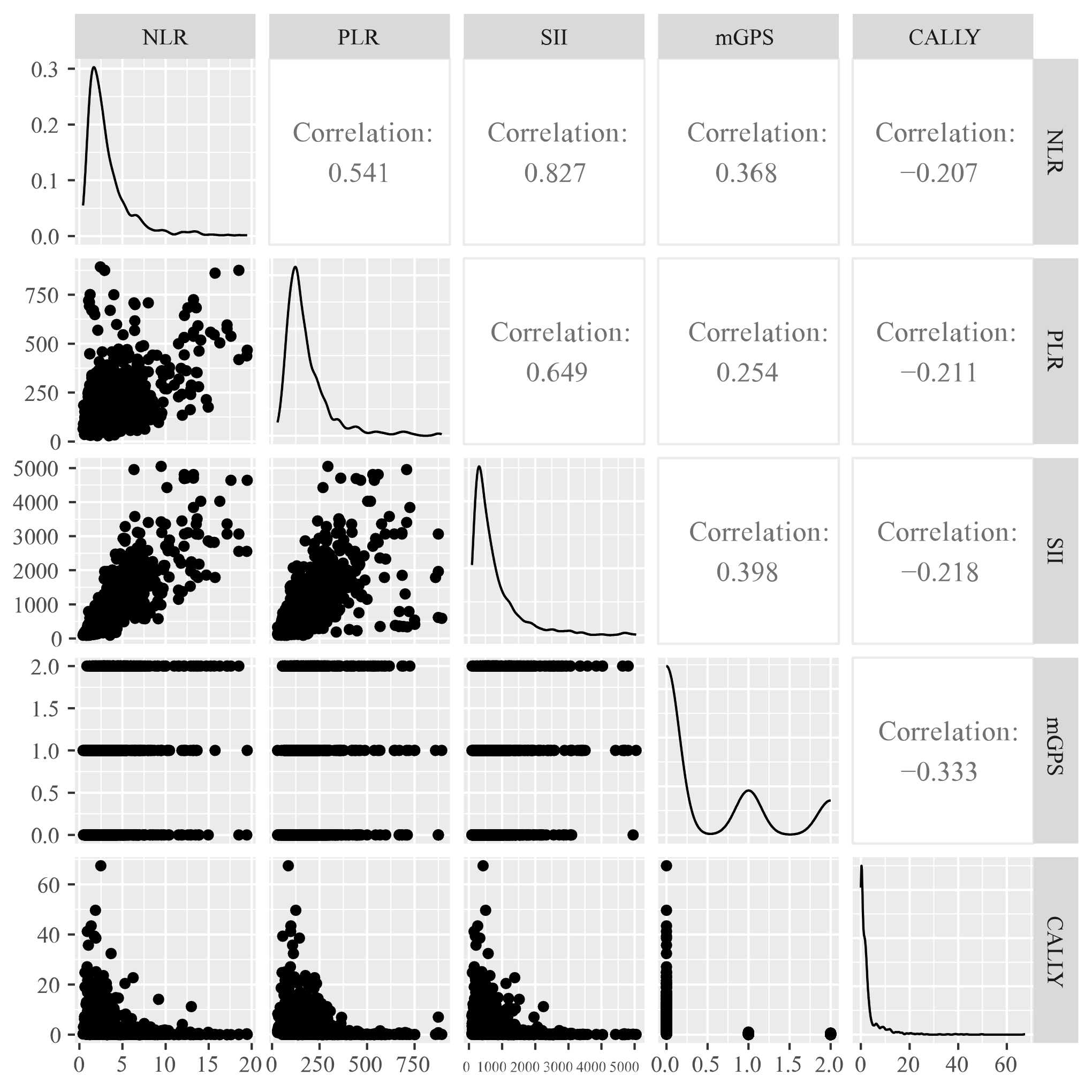


**Supplemental Figure 4.** Correlation analysis of CALLY index with NLR, PLR, SII and mGPS.

Abbreviation: CALLY, C-reactive protein-albumin-lymphocyte; NLR, neutrocyte to lymphocyte ratio; PLR, platelet to lymphocyte ratio; SII, systemic immune inflammation index; mGPS, modified Glasgow prognostic score.


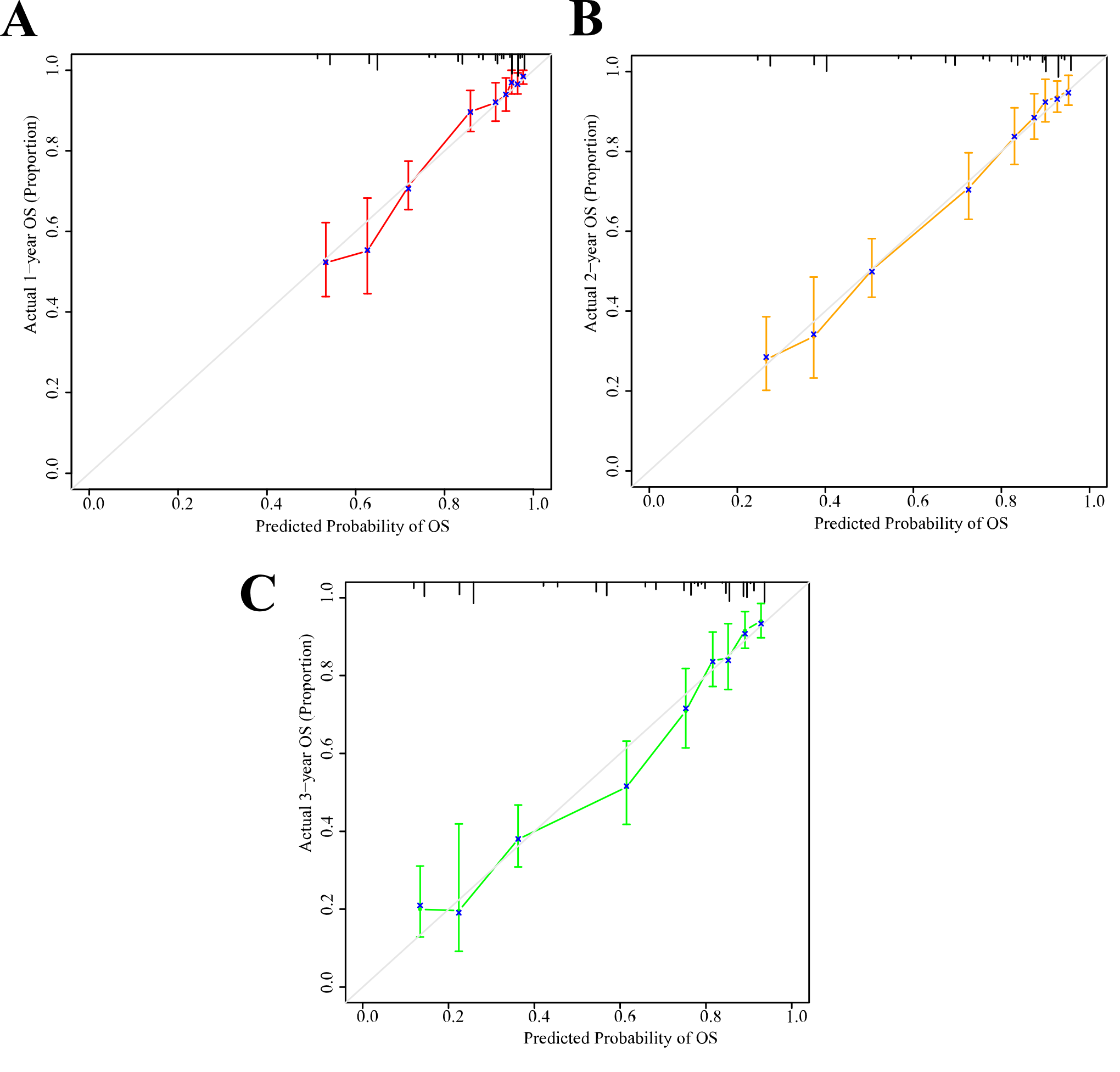


**Supplemental Figure 5.** The 1-, 2-, and 3- year calibration curves (A, B and C, respectively) of the nomogram model for predicting survival probability of patients with CRC.

Abbreviation: OS, overall survival; CRC, colorectal cancer.


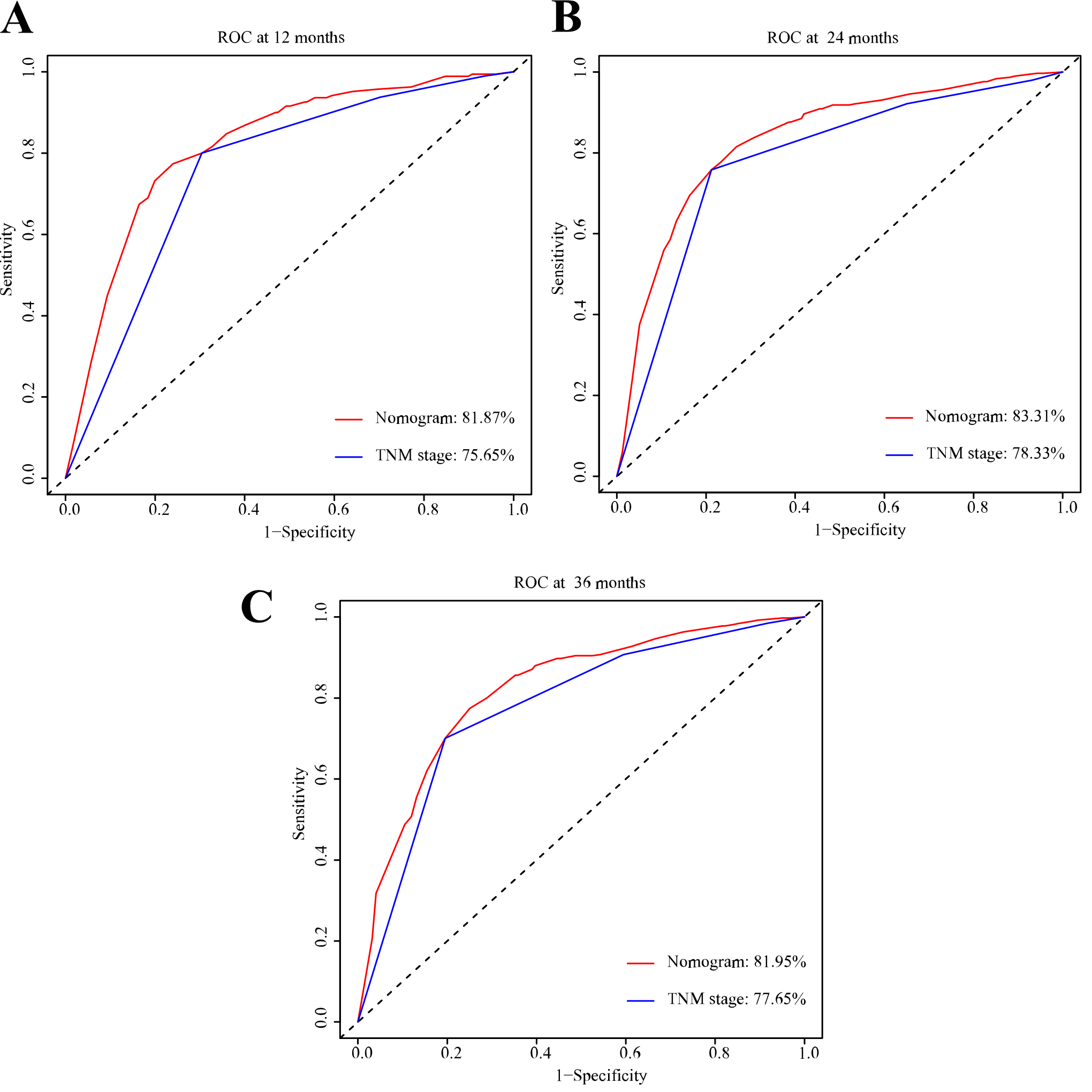


**Supplemental Figure 6.** The 1-, 2-, and 3- year AUCs (A, B and C, respectively) of the nomogram and the TNM stage in patients with CRC.

Abbreviation: ROC, receiver operating characteristic; AUC, area under the ROC curve; CRC, colorectal cancer.

**Supplemental Table 1. Criteria of mGPS.**

| Description | mGPS |
| --- | --- |
| CRP ≤ 10 mg/L and albumin ≥ 35 g/L | 0 |
| CRP ≤ 10 mg/L and albumin < 35 g/L | 0 |
| CRP > 10 mg/L and albumin ≥ 35 g/L | 1 |
| CRP > 10 mg/L and albumin < 35 g/L | 2 |

Notes:

Abbreviations: mGPS, modified Glasgow prognostic score; CRP, C-reactive protein.

**Supplemental Table 2. Associations between the CALLY index and OS in patients with CRC and survival time more than one year.**

| CALLY index | Model 1 *^a^* |  | Model 2 *^b^* |  | Model 3 *^c^* |  |
| --- | --- | --- | --- | --- | --- | --- |
|  | HR (95% CI) | *P* value | HR (95% CI) | *P* value | HR (95% CI) | *P* value |
| Continues | 0.90 (0.86, 0.95) | <0.001 | 0.91 (0.87, 0.96) | <0.001 | 0.92 (0.88, 0.96) | <0.001 |
| Low *^d^* | Reference |  | Reference |  | Reference |  |
| High *^d^* | 0.54 (0.41, 0.72) | <0.001 | 0.57 (0.43, 0.75) | <0.001 | 0.60 (0.45, 0.81) | 0.001 |
| Quartile 1 *^e^* | Reference |  | Reference |  | Reference |  |
| Quartile 2 *^e^* | 0.82 (0.57, 1.18) | 0.277 | 0.66 (0.45, 0.95) | 0.026 | 0.72 (0.49, 1.05) | 0.090 |
| Quartile 3 *^e^* | 0.83 (0.58, 1.19) | 0.308 | 0.72 (0.50, 1.04) | 0.078 | 0.80 (0.54, 1.19) | 0.274 |
| Quartile 4 *^e^* | 0.34 (0.22, 0.51) | <0.001 | 0.33 (0.22, 0.50) | <0.001 | 0.36 (0.23, 0.56) | <0.001 |
| *P* for trend | 0.74 (0.66, 0.84) | <0.001 | 0.73 (0.65, 0.83) | <0.001 | 0.76 (0.66, 0.86) | <0.001 |

Notes:

Abbreviations: CALLY, C-reactive protein–albumin–lymphocyte; OS, overall survival; CRC, colorectal cancer; HR, hazard ratio; CI, confidence interval; KPS, Karnofsky performance status score; PG-SGA, Scored Patient-Generated Subjective Global Assessment.

***^a^*** Model 1 was not adjusted for any covariates.

***^b^*** Model 2 was adjusted for sex, age, body mass index, and TNM stage.

***^c^*** Model 3 was adjusted for sex, age, body mass index, TNM stage, smoking status, alcohol consumption, KPS and PG-SGA.

***^d^*** Low: <1.47; High: ≥1.47.

***^e^*** Quartile 1: <0.27; Quartile 2: ≥0.27 and <1.35; Quartile 3: ≥1.35 and <2.82; Quartile 4: ≥2.82.

**Supplemental Table 3. C-indices of the CALLY index and other prognostic factors in patients with CRC.**

| Characteristics | C-indices (95% CI) | *P* value |
| --- | --- | --- |
| CALLY index | 0.668 (0.644, 0.693) |  |
| mGPS | 0.657 (0.632, 0.683) | <0.001 |
| NLR | 0.642 (0.615, 0.668) | 0.001 |
| SII | 0.632 (0.606, 0.657) | 0.001 |
| PLR | 0.629 (0.602, 0.655) | <0.001 |

Notes:

Abbreviations: C-indices, concordance indices; CRC, colorectal cancer; CI, confidence interval; CALLY, C-reactive protein–albumin–lymphocyte; mGPS, modified Glasgow prognostic score; NLR, neutrocyte to lymphocyte ratio; SII, systemic immune inflammation index; PLR, platelet to lymphocyte ratio.

**Supplemental Table 4. The C-indices of the nomogram and the TNM stage in patients with CRC.**

| Characteristics | C-indices (95% CI) | *P* value |
| --- | --- | --- |
| Nomogram | 0.767 (0.744, 0.791) |  |
| TNM stage | 0.727 (0.704, 0.750) | <0.001 |

Notes:

Abbreviations: C-indices, concordance indices; CRC, colorectal cancer; CI, confidence interval.
